# EMD-based data augmentation method applied to handwriting data for the diagnosis of Essential Tremor using LSTM networks

**DOI:** 10.1038/s41598-022-16741-y

**Published:** 2022-07-27

**Authors:** José Fernando Adrán Otero, Karmele López-de-Ipina, Oscar Solans Caballer, Pere Marti-Puig, José Ignacio Sánchez-Méndez, Jon Iradi, Alberto Bergareche, Jordi Solé-Casals

**Affiliations:** 1 Faculty of Computer Science, Multimedia and Telecommunications, Open University of Catalonia, Barcelona, 08080 Spain; 2 Data and Signal Processing Research Group, University of Vic-Central University of Catalonia, Vic, 08500 Spain; 3 EleKin Research Group, System Engineering and Automation Department, University of the Basque Country UPV/EHU, Donostia, Spain; 4 Department of Psychiatry, University of Cambridge, Cambridge, CB2 3EB UK; 5 Department of Business Management, Universidad del País Vasco/Euskal Herriko Unibertsitatea (UPV/EHU), EleKin Research Group, Donostia, Spain; 6 Neurodegenerative Disorders Area, Biodonostia Health Research Institute, Donostia, Spain

**Keywords:** Biomedical engineering, Movement disorders

## Abstract

The increasing capacity of today’s technology represents great advances in diagnosing diseases using standard procedures supported by computer science. Deep learning techniques are able to extract the characteristics of temporal signals to study their patterns and diagnose diseases such as essential tremor. However, these techniques require a large amount of data to train the neural network and achieve good results, and the more data the network has, the more accurate the final model implemented. In this work we propose the use of a data augmentation technique to improve the accuracy of a Long short-term memory system in the diagnosis of essential tremor. For this purpose, the multivariate Empirical Mode Decomposition method will be used to decompose the original temporal signals collected from control subjects and patients with essential tremor. The time series obtained from the decomposition, covering different frequency ranges, will be randomly shuffled and combined to generate new artificial samples for each group. Then, both the generated artificial samples and part of the real samples will be used to train the LSTM network, and the remaining original samples will be used to test the model. The experimental results demonstrate the capability of the proposed method, which is compared to a set of 10 different data augmentation methods, and in all cases outperforms all other methods. In the best case, the proposed method increases the accuracy of the classifier from 83.20% to almost 93% when artificial samples are generated, which is a promising result when only small databases are available.

## Introduction

Essential tremor (ET) is a disorder of the nervous system that causes involuntary and rhythmic movements. The tremor is at a frequency between 4 and 12 Hz and only occurs when the muscle is strained. The amplitude of the tremor is not constant throughout the course of the disease,since it varies as the patient ages. Although it can arise in any part of the body, this tremor appears most frequently in the hands, and especially when the subject performs simple everyday tasks, such as eating, drinking or tying their shoelaces. Usually, it is not a serious condition, but can become so if it is severe and can be mistaken for Parkinson’s disease^1^. ET is a worldwide disease which is more common than Parkinson’s disease (PD), approximately twenty times more according to^2^. The prevalence of this motor disorder is estimated to be between 0.3 and 4.0% in the Western world. It affects men and women in more or less equal proportions, with no gender differences being found. Its incidence is approximately 23.7 per 100,000 individuals per year and a large majority of cases, between 50 and 70%, are genetic in origin^2^.

In order to palliate and manage the symptoms, it is extremely important to be able to make a clinical diagnosis at the earliest manifestations of the disease. Early diagnostic techniques and the development or acquisition of usable clinical markers have advanced significantly in recent years, although these tests can be very expensive, such as the use of Single Photon Emission Computed Tomography (SPECT), and therefore not widely available in routine clinical practice^3^. Despite the usefulness of these biomarkers, the expense and technology requirements make it unfeasible to use these tests for all patients with motor conditions. It is in this context that intelligent non-invasive diagnostic techniques can be very useful tools for the early diagnosis of neurodegenerative disorders. Among these techniques, handwriting analysis using Artificial Intelligence (AI) is a powerful option. Non-technical staff in the patient’s environment could use this technique without interfering with the patient’s daily life, as handwriting analysis is not considered stressful by most patients. In addition, this technique has a very low cost and its requirements in terms of infrastructure or medical equipment are very modest, providing information easily, quickly and cheaply. Currently, the Archimedes spiral is the gold standard test in clinical diagnosis^4^.

Different studies, such as^5–7^, have focused on the automatic analysis of handwritten data to determine the patterns that allow an accurate diagnosis of this disease. In this work we will study the use of a deep neural network based on LSTM^8,9^ to perform this diagnosis, so that the neural network itself will extract the main patterns from the data.

On the other hand, it is common for a Deep Learning system to use hundreds or thousands of samples to train the system, given that the number of parameters of the model is very high. In our case, we only have a total of 51 samples. With small datasets, overfitting is a problem to be taken into account. Overfitting occurs when the system becomes too specialised with training data and is unable to successfully classify new test data, thus losing the ability to generalise. To reduce or avoid this problem, the input dataset should be larger.

Data augmentation (DA) refers to the process of synthesising new data from existing data. Applying various transformations to the available data to synthesise new data is one approach to deal with the issue of insufficient data^10^, but in this work we will use a method based on a decomposition–recombination technique to generate artificial samples that has been tested on one-dimensional data (time series) but, to our knowledge, has never been used before in this scenario, dealing with 2D data (hand-drawn spirals). We will compare our proposed method with a set of 10 methods commonly used in time series data augmentation. As will be seen in the experiments, our method clearly outperforms all others, proving to be a very good candidate for this problem.

## Methods

One of the challenges of this project is having only a small number of subjects (samples) for training an LSTM model. To reduce this problem, a data augmentation method will be used, which will extend the number of samples used by more than 500%, increasing the variability of the input data, and thus reducing over-fitting.

The different steps to generate artificial random samples for training the model are explained below. First, the working scenario is presented and the samples are divided in two groups: ET (essential tremor patients) and CT (control subjects). Then, using the multivariate Empirical Mode Decomposition (mEMD)^11,12^, the frequency decomposition of all samples in the dataset is performed. This frequency decomposition generates the so-called Intrinsic Mode Functions (IMFs). Finally, using only the IMFs of the subjects selected for training, the new random samples are generated.

### Scenario

The analysis and diagnosis of the patients will be carried out using the dataset named BIODARW. This data was firstly presented in^2^ and has been used in previous works^5–7^ to analyze and classify samples (drawings) from ET patients and controls. As stated in^5–7^, this study was carried out in accordance with the recommendations of the Ethics Committee of the Donostia University Hospital (San Sebastian, Spain), which approved the protocol. All subjects gave written informed consent in accordance with the Declaration of Helsinki.

The participants in the study performed, among other exercises, the test of drawing the Archimedes’ Spiral^4^ with both hands, using a digitizing tablet to acquire the samples.

In order to compare our results with the previous mentioned works^5–7^, we will use the same subset, BIODARWO, which consists of 51 samples: 24 samples for the ET group and 27 samples for the control group. Please, refer to^5–7^ for more details on the dataset.

The data available in this database was obtained from a Wacom 4 digitizing tablet at a sampling frequency of 100 Hz. Each sample of data is stored in a matrix in which each column contains:

1. X Coordinate
2. Y Coordinate
3. Time Stamp
4. Status (0,1) (1 when the pen touches the paper)
5. Azimuth
6. Angular Altitude
7. Pressure

We will only work with the X and Y coordinates, as it was done in^7^ for two main reasons: (1) any digital tablet can obtain these parameters, but maybe not the others (angles, pressure, etc.); (2) X and Y coordinates carry enough information to diagnose essential tremor, as demonstrated in^7^.

### Empirical mode decomposition

EMD is based on a method that allows decomposing the different time signals into a finite number of the so called Intrinsic Mode Functions (IMFs)^11^. Each IMF represents a non-linear oscillation of the original signal, which can be reconstructed by summing all the IMFs of the signal and its residual (the IMF corresponding to the linear trend). All the IMFs have to fulfill the following two conditions:

1. The number of maximums has to be the same as the number of zero crossings, or at least they have to differ by only one.
2. For any sample, the mean value between the envelope of the local maxima and that of the local minima must be zero.

The algorithm of this decomposition method consists in rewriting a real-valued signal *x*(*t*) as:

1 $$\begin{aligned} x(t) = \sum _{n} x_n(t)+r(t) \end{aligned}$$

where $x_n(t)$ are the so-called IMFs and *r*(*t*) is the residue signal.

The iterative process to obtain them is described below:

1. Define $$s(t)=r_{n-1}(t)$$. Initialize $$n = 1$$, $$r_0(t) = x(t)$$.
2. Extract the n-th IMF as follows:
  - Identify all the local maximum and minimum of *s*(*t*).
  - Interpolate between the maximum (minimum) to obtain the upper envelope (lower envelope).
  - Obtain the local mean *m*(*t*) by taking the mean of both envelopes.
  - Obtain an IMF candidate by subtracting the local mean *m*(*t*) to the signal *s*(*t*): $h(t)=s(t)-m(t)$.
  - If *h*(*t*) does not comply with the two conditions to become an IMF then go to step 2 with $s(t)=h(t)$
3. If *h*(*t*) satisfy the IMF conditions, then:
  2 $$\begin{aligned} x_n(t)=h(t) \text { and } r_n(t)=r_{n-1}(t)-x_n(t) \end{aligned}$$
4. If $r_n(t)$ is a monotone function, or does not have enough extreme points to calculate the upper and lower envelopes, then $x_n(t)$ is the last IMF function of *x*(*t*) and the decomposition ends.
5. Otherwise, set $s(t) = r_n(t)$ and iterate from step 2 to obtain the next IMF.

In order to simultaneously decompose X and Y coordinates for each sample and for all the subjects, the multivariate EMD (mEMD)^12^ algorithm was used. In this way, the maximum and minimum envelope will be the same for all subjects and coordinates, and the number of IMFs will be the same for all of them. The decision to use mEMD instead of EMD was made to overcome the problem of having a different number of IMFs depending on the subject, due to the intrinsic data-driven characteristics of the EMD algorithm. This effect induced a desynchronisation of the weights, which caused the artificial samples to work improperly, clearly deteriorating the accuracy of the classification system.

**Figure 1:**
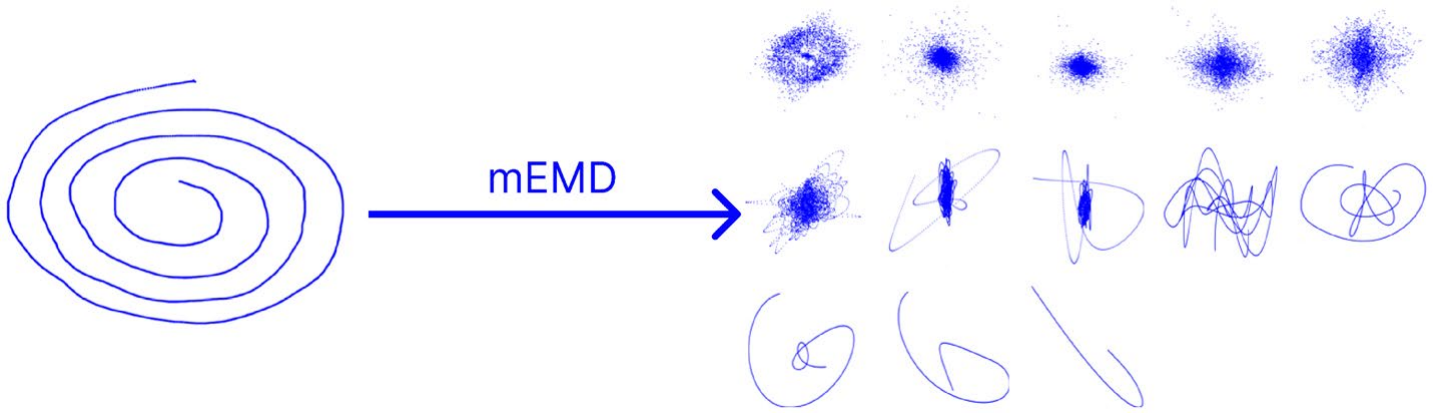
Extraction of IMFs through multivariate empirical mode decomposition.

The decomposition process is presented in Fig. 1, corresponding to a control subject, in which the original spiral is observed on the left, and the obtained IMFs, from high to low frequency, are are shown in the right.

### New artificial samples

The method to create artificial samples is based on previous works in which this method was first presented and used on EEG data for decreasing the calibration step in a BCI application^13^, and then used in^14^ as a data augmentation technique to train a deep learning classifier, also on EEG data.

To our knowledge, this is the first time this method has been used with handwritten data to create artificial drawings of Archimedes’ spirals.

**Figure 2:**
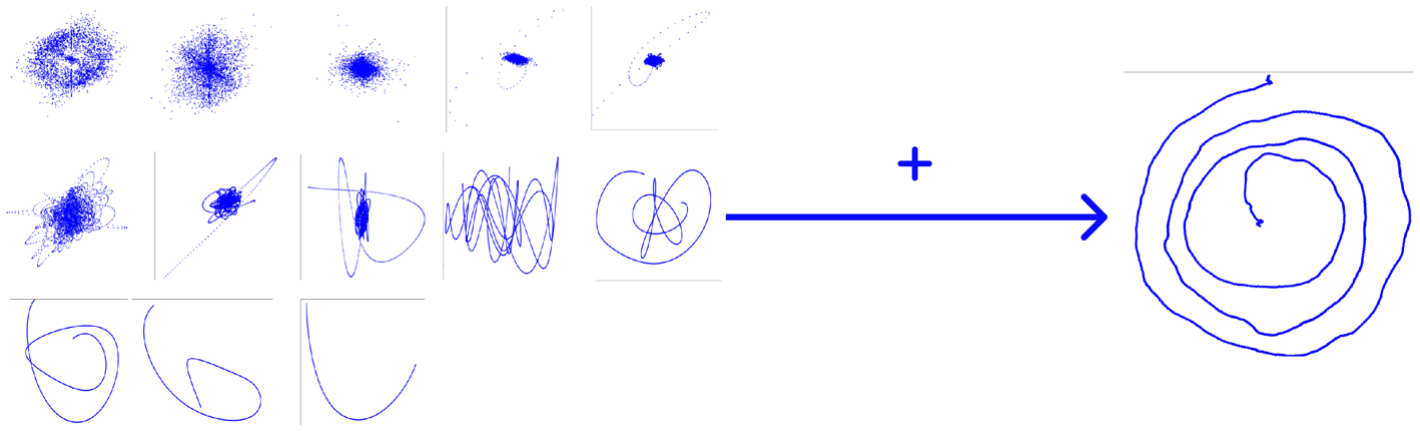
The process of generating a new sample using different IMFs of random subjects of the same class.

Once the IMFs for each subject and coordinate (X and Y) have been obtained, and the group of training subjects has been selected, artificial samples can be generated. The strategy consists of:

1. Define the number of artificial samples to be generated, per each group of subjects (ET and CT).
2. Randomly select the IMFs to be used to create each artificial subject. This has to be done separately for each group.
3. Generate the new samples by summing the selected IMFs, obtaining the new samples, as shown in Fig. 2.

**Figure 3:**
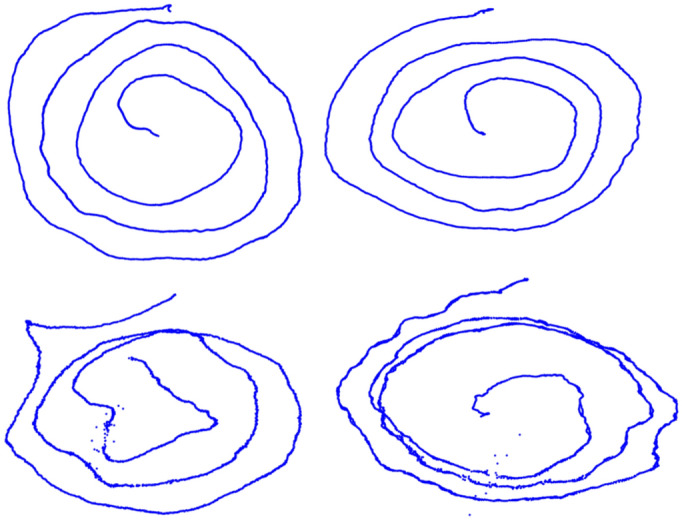
Artificial frames generated with the proposed method. On the top, two examples of artificial CT samples; On the bottom, two examples of artificial ET samples.

Table 1 summarizes the procedure, showing how to create an artificial subject by adding one IMF of each band, after decomposing *N* subjects, and Fig. 3 depicts some examples of artificial Archimedes’ spirals generated with this method. Because we use information of some subjects (their IMFs) to create the artificial samples, these real subjects will be part of the training dataset, together with the artificial ones. The remaining (non-used) subjects will conform the test dataset.

**Table 1:** Generation of an artificial subject. The IMFs in bold, one of each order and coming from several subjects, are added together to generate an artificial subject.

	IMF 1	IMF 2	IMF 3	IMF 4	IMF N
Subject 1	**S1-IMF 1**	S1-IMF 2	S1-IMF 3	S1-IMF 4	**S1-IMF N**
Subject 2	S2-IMF 1	S2-IMF 2	S2-IMF 3	**S2-IMF 4**	S2-IMF N
Subject 3	S3-IMF 1	**S3-IMF 2**	S3-IMF 3	S3-IMF 4	S3-IMF N
Subject 4	S4-IMF 1	S4-IMF 2	S4-IMF 3	S4-IMF 4	S4-IMF N
Subject 5	S5-IMF 1	S5-IMF 2	**S5-IMF 3**	S5-IMF 4	S5-IMF N
Subject N	SN-IMF 1	SN-IMF 2	SN-IMF 3	SN-IMF 4	SN-IMF N
New subject	S1-IMF 1	S3-IMF 2	S5-IMF 3	S2-IMF 4	S1-IMF N

### Other data augmentation methods

To compare our data augmentation method with other methods, we briefly describe here a set of 10 methods that will also be used with the handwriting data. These methods can be grouped in: (1) magnitude domain transformation methods; (2) time domain transformations methods; (3) frequency domain mixing methods, and (4) generative models methods. All these algorithms are described and analysed in^15^ and the code, provided by the authors, can be download here: https://github.com/uchidalab/time_series_augmentation. We refer the reader to^15^ and references therein for more details on these methods.

The Magnitude domain transformation methods were: Jittering, Magnitude warping, Rotation and Scaling. For Jittering, Gaussian noise is added to the time series. For the Magnitude warping, the magnitudes of the time series are multiplied by a warping amount determined by a cubic spline line with four knots at random locations and magnitudes. For Rotation, because we have univariate time series, flipping is used, in which 50% of the patterns of the training set are inverted at random for each data augmentation set. Scaling method multiplies training set time series with random scalars from a Gaussian distribution. In this way, the time series are scaled by a single multiplier for all time steps.

The Time domain transformations methods were: Permutation, Window slicing, Time warping and Window warping. In the Permutation method, two to five equal sized segments were permuted. Window slicing crops the time series to 90% of the original length. The starting point of the window slice is chosen at random and interpolation is used to keep the original length of the time series. Time warping uses a warping path defined by a smooth cubic spline-based curve with four knots of random magnitudes. Window Warping selects a random window, that is 10% of the original time series length and warps the time dimension by 0.5 times or 2 times.

The Frequency domain mixing method was SuboPtimAl Warped time series geNEratoR (SPAWNER), which is a pattern mixing data augmentation method that suboptimally averages two intra-class randomly selected patterns. In addition, noise is also added to further transform the data.

Finally, the Generative model method was a Conditional Generative Adversarial Network (cGAN)^16^. cGANs are similar to GANs but they use the label of the samples as a parameter during the training process, to generate data that belong to specific categories. In that case we used directly the Matlab implementation of cGAN.

### LSTM network

An LSTM network is a type of recurrent neural network (RNN) capable of learning long-term dependencies between time steps in a sequence of data. When a traditional RNN is trained by backpropagation, the long-term gradients that are backpropagated can vanish due to the computations involved in the process. The contrary effect can also happens, and in some situations the long-term gradients can explode. Therefore, traditional RNN are not useful when long-term dependencies are involved.

**Figure 4:**
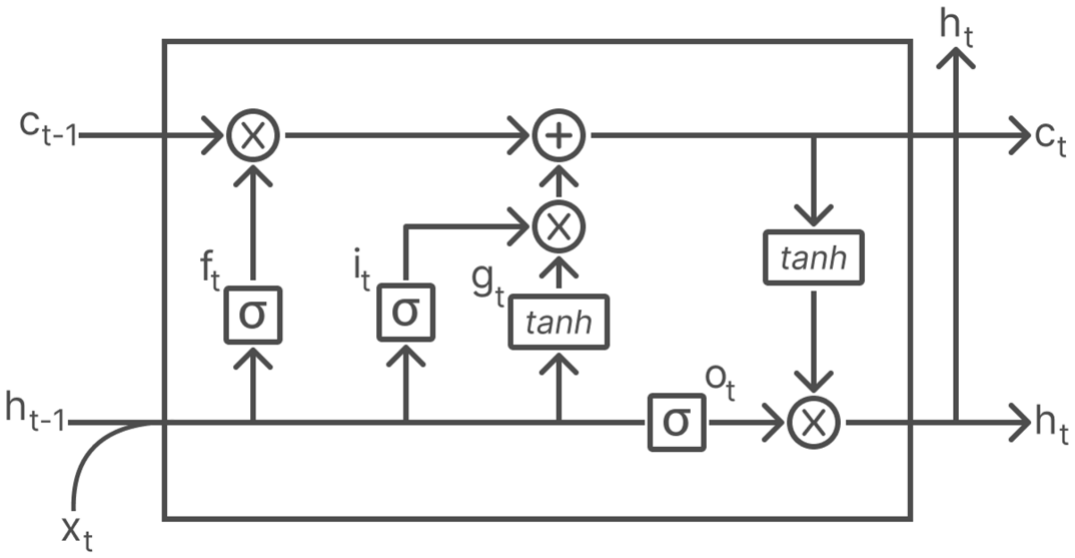
LSTM cell.

LSTM networks overcome the vanishing gradient problem and work very well for data sequences. Internally, as represented in Fig. 4, an LSTM layer at a given instant *t* consists of the hidden state, the vector $h_t$ and the state of the cell, the vector $c_t$. The hidden state at time step *t* is also the output of the LSTM layer for this time step. The cell state contains the information learned in the previous time steps. At each time step, the layer adds information to the cell state or removes it. The layer controls these updates through the following gates: (1) the entry gate, $i_t$, which controls the level of the cell state update; (2) the forget gate, $f_t$, which controls the level of the cell state reset (forget); (3) the cell candidate, $g_t$, which adds information to the cell state; and (4) the exit gate, $o_t$, which controls the level of the cell state added to the hidden state. Note than in Fig. 4 the symbols $\sigma$ and *tanh* represent the sigmoid and hyperbolic tangent activation functions, respectively.

When the information of sequence $t+1$ is available at processing time *t*, i.e., when we have the raw sequence from the beginning of its processing, as is the case here, we can combine two LSTM structures, each of them processing the sequence in different directions, one the forward direction and the other one in the backward direction. In this case the layer is called biLSTM and it combines the information in both directions^9^. One of the most important parameters of this type of network is the number of hidden nodes. To make an LSTM networks deeper, extra LSTM layers can be added. To prevent overfitting, dropout layers are included after LSTM layers^17^.

In our case we built the model using 14 layers, operating in classification mode. The layers are the following:

- Layer 1: Input sequence layer where the sequence is represented by two features (the X and Y coordinates) of the points that define the spiral going from one end to the other.
- Layer 2: biLSTM with a total on 52 hidden units.
- Layer 3: Dropout layer of 20%.
- Layer 4: biLSTM with a total on 40 hidden units.
- Layer 5: Dropout layer of 20%.
- Layer 6: biLSTM with a total on 25 hidden units.
- Layer 7: Dropout layer of 20%.
- Layer 8: biLSTM with a total on 20 hidden units.
- Layer 9: Dropout layer of 20%.
- Layer 10: biLSTM with a total on 15 hidden units.
- Layer 11: Dropout layer of 20%.
- Layer 12: Fully Connected Layer, that will act as a convergence layer on which the number of output classes to be obtained is identified, i.e. it matches the number of groups identified at the input (2 in our case, control group and ET group)
- Layer 13: Softmax Layer. This layer, using the input from the previous layer, assigns probabilities to the different classes obtained from the previous layer.
- Layer 14: Classification Layer, which provides the final output of the model, according to the output of the previous layer, identifying whether the input data (X and Y coordinates of an spiral) is from a control subject or an essential tremor subject.

**Figure 5:**
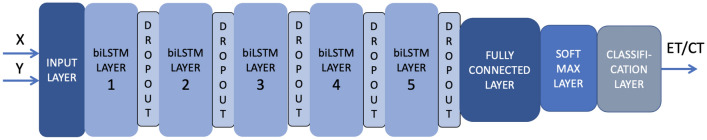
Structure of the LSTM used as a classifier.

The final classifier based on the LSTM model is represented in Fig. 5. This model was coded using Matlab, and optimized using the well known adaptive moment estimation algorithm (Adam), that usually works properly with time series signals. It was trained with 25 epochs on a mini-batch size of 48 samples per epoch, shuffling on every epoch the order of the samples to get more variability to the training model. The learning rate was established at 0.001 as initial rate, with a learn drop factor of 0.9 each 10 epochs. Finally, for the regularization parameters, L2 Regularization value was established at $1 \cdot 10^{-4}$, and the Dropout factor between each layer at 0.2.

### Model evaluation

To evaluate the results, the accuracy (ACC), the sensitivity or true positive rate (SEN) and the specificity or true negative rate (SPE) will be used, calculated as follows:

3 $$\begin{aligned}&ACC = \frac{TP+TN}{ TP+TN+FP+FN} \end{aligned}$$

4 $$\begin{aligned}&SEN = \frac{TP}{TP+FN} \end{aligned}$$

5 $$\begin{aligned}&SPE = \frac{TN}{TN+FP} \end{aligned}$$

where TP, TN, FP, and FN are, respectively, the true positive, true negative, false positive and false negative values of the confusion matrix. The positive case corresponds to the ET subjects while the negative case to the CT ones.

## Experiments and results

To validate the use of the proposed strategy as a DA method to create artificial samples for a better diagnosis of patients with ET, two experiments have been proposed:

- In the first experiment, 7 subjects from each group were randomly selected, and the model was trained using these subjects with different number of artificial samples: 0, 5, 50 and 100 artificial samples per group. This experiment allowed to quantify the effect of the artificial samples, and to identify which subjects from the test dataset were wrongly classified.
- In the second experiment, several training runs were performed with 10 repetitions for each, using the same parameters for the LSTM model but with a random selection of subjects. Therefore, the experiment allowed us to analyse the results in terms of accuracy, sensitivity and specificity, calculating the mean and standard deviation for each case.

### Training with fixed subjects

This experiment was carried out with 0, 5, 50 and 100 artificial samples generated for each group, using the same original input samples for training. The subjects for the training step were:

- Control subjects: 1, 4, 6, 10, 13, 16 and 21
- ET subjects: 1, 3, 7, 13, 16, 18 and 22

**Figure 6:**
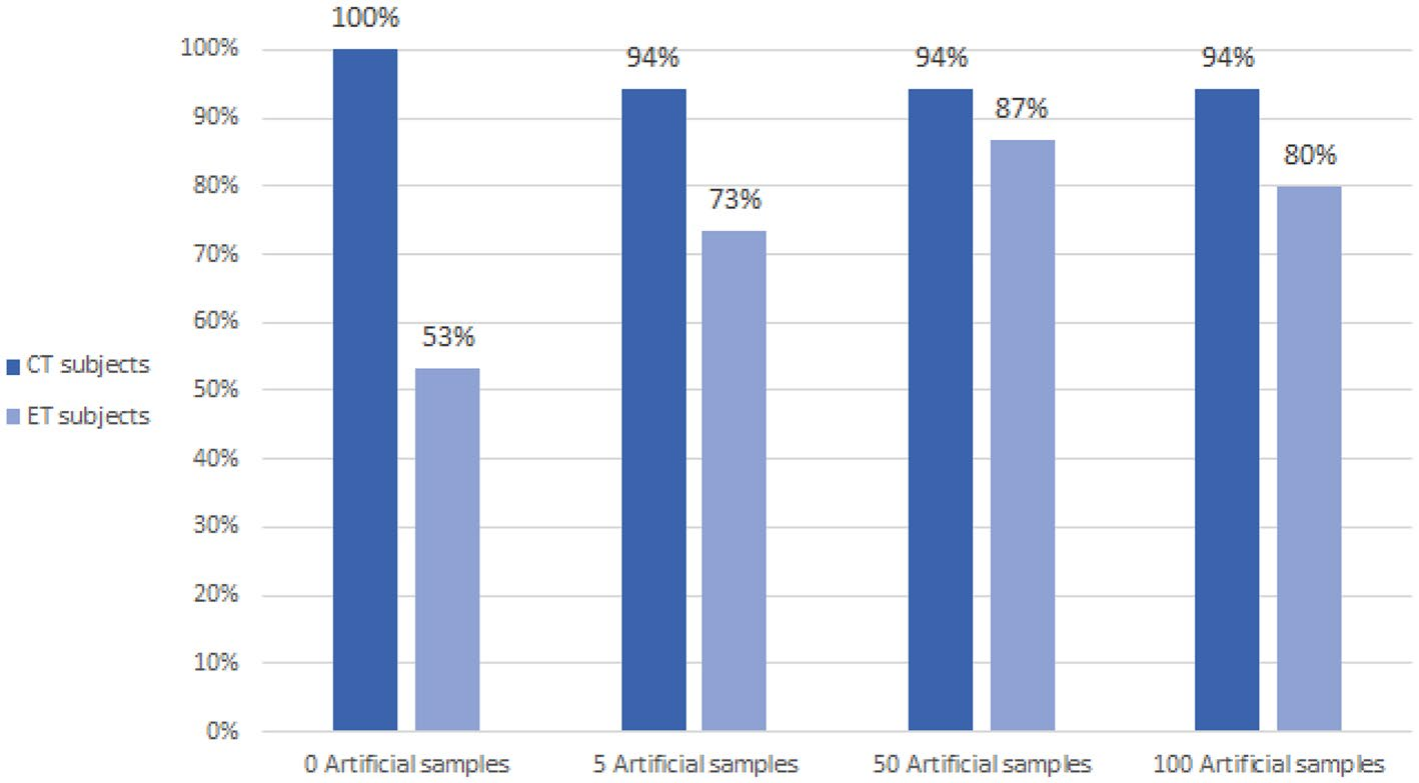
True negative rate (CT subjects) and true positive rate (ET subjects) depending on the number of artificial frames for the same subjects in the training step.

The model was trained with these subjects plus the artificial data generated by combining IMFs from them. Therefore, the changes in the accuracy of the results were determined by the number of artificial samples generated in each case. Results for the true negative rate (CT subjects) and true positive rate (ET subjects) are depicted in Fig. 6, depending on the number of artificial frames for the same subjects in the training step.

**Table 2:** Results (in terms of accuracy, sensitivity and specificity) of the diagnosis for each case, when using different number of artificial samples.

	ACC (%)	SEN (%)	SPE (%)
0 artificial samples	80.00	53.00	**100.00**
5 artificial samples	85.71	73.33	95.00
50 artificial samples	**91.43**	**86.67**	95.00
100 artificial samples	88.57	80.00	95.00

﻿Best cases are highlighted in bold.

Numerical results for the accuracy, sensitivity and specificity are presented in Table 2. Detailed results for all the subjects used in the test step are shown in Table 3, which contains the results for the 4 different scenarios tested in this experiment (0, 5, 50 and 100 artificial samples per group).

**Table 3:** Detailed results of the number of subjects correctly and incorrectly classified for each class, depending on the number of artificial samples generated.

	0 Art. samples	5 Art. samples	50 Art. samples	100 Art. samples
**CT subjects**				
OK (TN)	20	19	19	19
NOK (FP)	0	1	1	1
**ET subjects**				
OK (TP)	8	11	13	12
NOK (FN)	7	4	2	3

### Training with random subjects

Since the results obtained when training a model fluctuate if it is executed several times and for different input data, in this case we repeated 10 times the same experiment, with a random selection of subjects used for the training step, and with 0, 5, 50, 100 or 250 artificial samples of each group. Moreover, the number of real subjects in the training step was explored considering 10%, 30% or 50% of the original subjects for training. In each case, the artificial samples were generated using the IMFs of the selected original subjects.

**Figure 7:**
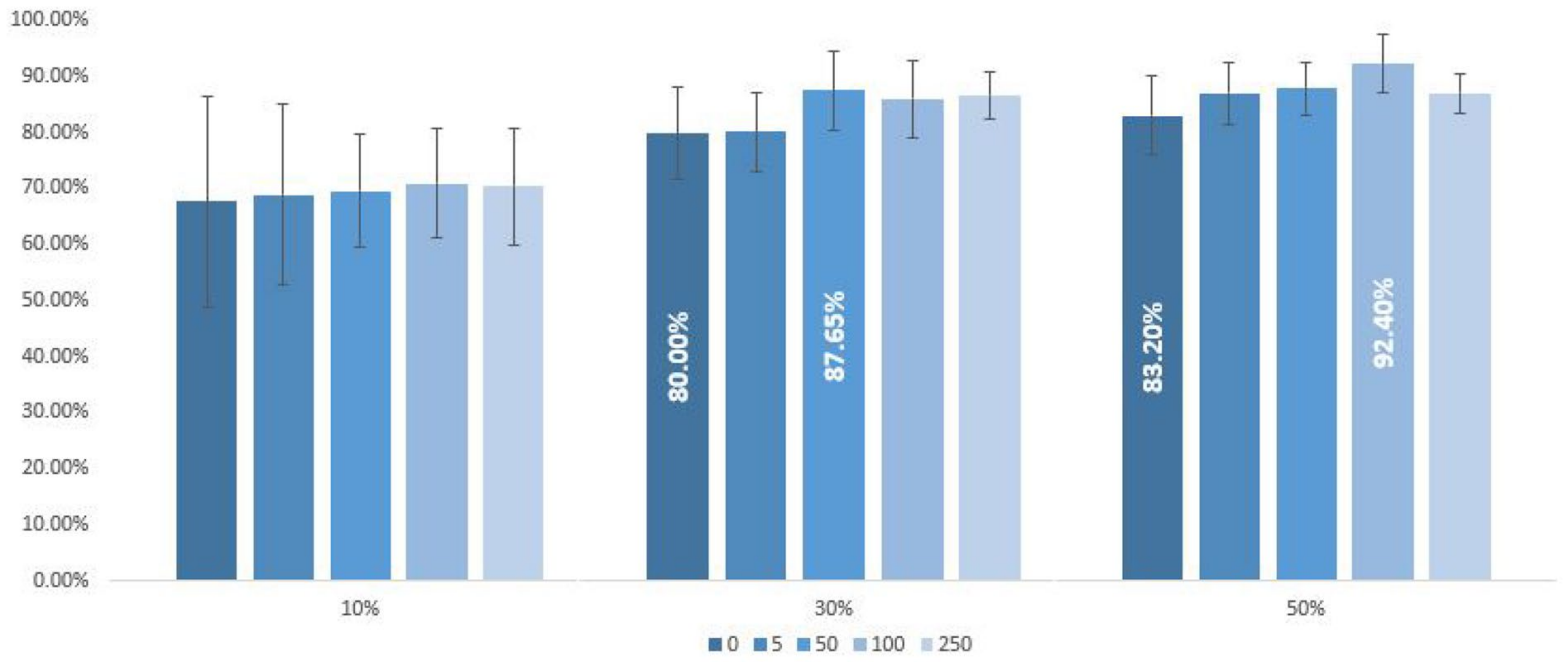
Accuracy (%) of the testing diagnosis depending on the number of artificial samples and the percentage of original samples used for training. Each group of 5 bars correspond to a different percentage of original samples used for training. The number of artificial samples in each case is represented by the different colours of the bars. The best and the second-best cases are indicated with the corresponding value of accuracy.

Figure 7 shows the accuracy of these experiments, while Table 4 show the numerical results for the accuracy, the sensitivity and the specificity, depending on the number of artificial samples and for each percentage of original data used in the training set.

**Table 4:** Accuracy, sensitivity and specificity of the testing diagnosis depending on the number of artificial samples and the percentage of original samples used for training.

	% of samples for training		
	10%	30%	50%
**ACC (%)**			
0 artificial samples	67.73	80.00	83.20
5 artificial samples	68.86	80.29	87.20
50 artificial samples	69.55	**87.65**	88.00
100 artificial samples	**70.91**	86.18	**92.40**
250 artificial samples	70.45	86.76	87.20
**SEN (%)**			
0 artificial samples	55.50	81.33	68.18
5 artificial samples	55.50	76.67	78.18
50 artificial samples	**66.50**	**88.00**	85.45
100 artificial samples	62.50	85.33	**93.64**
250 artificial samples	54.00	86.00	83.64
**SPE (%)**			
0 artificial samples	77.92	78.95	**95.00**
5 artificial samples	80.00	83.16	94.29
50 artificial samples	72.08	**87.37**	90.00
100 artificial samples	77.92	**87.37**	91.43
250 artificial samples	**84.17**	**87.37**	90.00

Best cases are highlighted in bold.

### Comparison with other data augmentation methods

**Figure 8:**
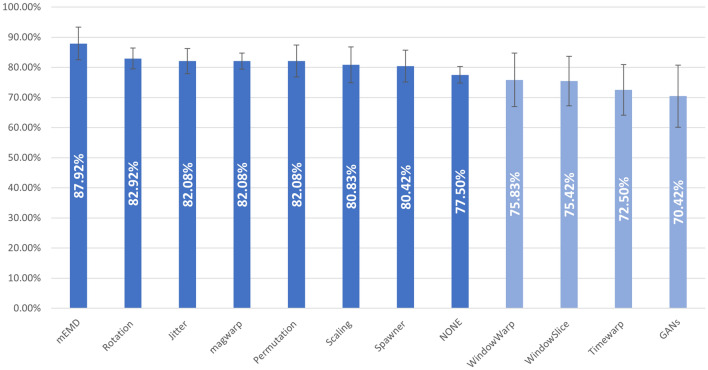
Mean accuracy (%) for all the DA methods and for the case of no artificial data added (NONE). In all the experiments (except NONE), 100 artificial samples per group were generated with the same 50% of the real data. The light blue bars correspond to the methods that worsened the average accuracy compared to the NONE method.

To validate the proposed method, we performed a set of experiments using the 10 different DA methods presented in “Other data augmentation methods”. This experiment was similar to the first experiment detailed in “Training with fixed subjects”, in which the same 50% of subjects, selected at random, were used as training set, and the rest were used for testing. From the training set, 100 artificial samples per group were generated with each one of the DA methods. Then, the classifier was trained 10 times, so the mean value and the standard deviation of the accuracy, the sensitivity and the specificity are reported. Moreover, the model was also trained using only real data, without data augmentation. This method is referred as NONE. Figure 8 depicts the accuracy of this experiment. The methods are organized according its performance. Our proposed method, based on mEMD, is the best one, while NONE is the eight algorithm. The values of the experiment are shown in Table 5.

**Table 5:** Mean and standard deviation of the accuracy, sensitivity and specificity for the proposed method based on mEMD, plus 10 data augmentation methods and NONE (no data augmentation).

	ACC (%)	SEN (%)	SPE (%)
mEMD	**87.92** ± 5.42	**87.27** ± 6.03	88.46 ± 7.88
Rotation	82.92 ± 3.46	79.19 ± 8.18	86.15 ± 10.77
Jitter	82.08 ± 4.19	78.18 ± 10.12	85.38 ± 6.39
Magwarp	82.08 ± 2.67	70.00 ± 7.10	**92.31** ± 4.87
Permutation	82.08 ± 5.29	81.82 ± 9.96	82.31 ± 6.01
Scaling	80.83 ± 5.95	77.27 ± 9.32	83.85 ± 8.03
Spawner	80.42 ± 5.29	71.82 ± 6.36	87.69 ± 7.05
NONE	77.50 ± 2.76	66.36 ± 5.82	86.92 ± 3.53
WindowWarp	75.83 ± 8.90	72.73 ± 7.04	78.46 ± 14.92
WindowSlice	75.42 ± 8.22	80.91 ± 9.49	70.77 ± 13.23
TimeWarp	72.50 ± 8.37	63.64 ± 4.07	80.00 ± 16.21
GANs	70.42 ± 10.28	68.18 ± 4.55	72.31 ± 17.94

The methods are listed from highest to lowest accuracy.

﻿Best cases are highlighted in bold.

## Discussion

Two experiments have been proposed to test the effectiveness of the use of artificial samples using the proposed method based on a decomposition–recombination strategy, and in both cases the results with artificial samples have clearly outperformed the results obtained without artificial samples.

In the first experiment (Table 2) the best case is obtained when 50 artificial samples are added to each group. The accuracy in this case was 91.43%, with a sensitivity of 86.67%. The specificity for that case, even if reaching 95%, was lower than the one obtained with the original data. As can be seen in Table 3, without using artificial samples, all the subjects from the control group were perfectly classified as controls (100% specificity), while seven ET subjects were classified as controls (53% sensitivity). When using artificial sample, one of the subjects was always incorrectly classified as ET, which decreased the specificity to 95%, but only 2, 3 or 4 patients (depending on the number of artificial samples) were classified as controls, increasing the sensitivity up to almost 87%, which is an interesting result. This experiment showed that the use of 50 artificial samples per group improved the original results in accuracy from 80 to 91.43% and in sensitivity from 53 to 86.67%, while in specificity it decreased slightly from 100 to 95%, giving a better and more balanced result.

In the second experiment, we calculated the mean value of 10 experiments in different scenarios, in terms of the percentage of subjects used for training, and the number of artificial samples generated for each group.

Table 4 summarizes the results in terms of accuracy, sensitivity and specificity. As can be seen, artificial samples always allow for increased accuracy when creating artificial samples. The best accuracy is obtained with 100 artificial samples per group and when 50% of the real subjects are used for training, increasing the accuracy from 83.20 to 92.40% (in the following, we will refer to this case as the best case), followed by an accuracy of 88% when only 50 artificial samples per group are created. Almost 88% accuracy is obtained when 30% of the real subjects are used for training and 50 artificial samples per group are created (in the following, we will refer to this case as the second-best case). These results represent an improvement in accuracy of more than 9% and 7% for the best and second-best cases respectively, due to the effect of the artificial samples.

The sensitivity is also improved in these cases, from 68.18% (no artificial samples) to 93.64% for the best case, and from 81.33% (no artificial samples) to 88% in the second-best case. Finally, the specificity slightly decreases from 95% (no artificial samples) to 91.43% in the best case, and increases from 78.95% (no artificial samples) to 87.37% in the second-best case. All these results are summarized in Fig. 7, which allow to visualize the effect of increasing the percentage of real data used for training and at the same time the effect of the number of artificial data added in the training set. Note also that the artificial samples help to reduce the variance of the classifier, which is an interesting property already pointed out in^18^.

When comparing the proposed method with the other DA methods, we found that our method outperforms all methods in terms of accuracy and sensitivity, and only for specificity did one of the methods perform better. It’s interesting to note that our method based on mEMD increased the accuracy by about 10.5% (up to almost 88%), while the best other case (Rotation) only increased it by 5.5% (up to almost 83%). The other methods that obtained an accuracy over 80% where: Jittering, Magnitud warping and Permutation, all of them with a mean accuaracy of 82.08%; Scaling, with a 80.83%; and SPAWNER with a 80.42%. Without artificial samples, the mean accuracy was of 77.50%. The rest of the methods obtained a mean accuracy below that value, being cGAN the worst case. Note that the accuracy obtained by mEMD is in line with the result obtained in the first experiment, as reported in the last row of Table 2 (100 artificial samples).

We observed that only the DA methods based on amplitude manipulations improved the results. The fact that Window warping, Window slice and Time warping did not provide results superior to the NONE case is probably due to the type of manipulation performed on the signal. In all three cases, the temporal relationship between the samples is affected, so the tremor (or lack of tremor) is also affected. Finally, the fact that cGAN is not useful is probably due to the lack of real samples. GANs are deep learning models that need huge amounts of data to train the generator, but in this application (and in many medical applications), the database is small. Therefore, if there is not enough data available, the artificially generated data is not useful, decreasing the performance of the classifier.

Focusing on the proposed method, the best case, obtained when using 50% of the original samples for training plus 100 artificial samples, represents an interesting improvement compared to the case without artificial samples, but it is interesting to note that when using only 30% of the original samples for training and adding 50 or more artificial samples per group, the accuracy obtained is better than when using 50% of the original data without artificial samples. This is relevant because it indicates that mEMD-based artificial sample generation is useful when we have small datasets, where only a few samples are available to build the model. In this example, 15 original samples were used for training, to which the artificial samples generated per group were added. The new artificial samples contributed positively to the training of the LSTM model, improving its accuracy and controlling the over-fitting of the system.

**Figure 9:**
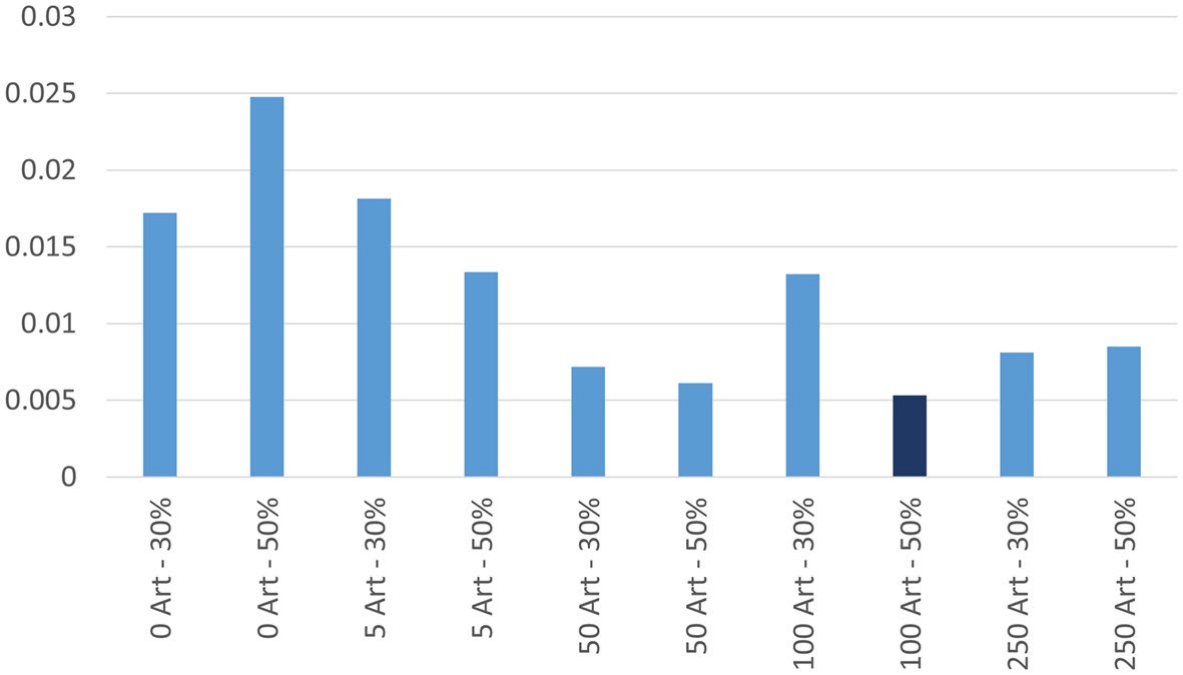
Difference in absolute value of the MCC and the Kappa indices. The smaller the difference, the more balanced the system will be. The best case is highlighted in dark blue.

To emphasise the fact that the proposed method is useful to increase accuracy but also to balance and improve sensitivity and specificity, we calculated Cohen’s Kappa and Matthews Correlation Coefficient (MCC). We used the same methodology presented in^19^ and calculated the difference in absolute value of the two indices. Graphical results are presented in Fig. 9. The smaller the difference, the more balanced the system will be.

**Figure 10:**
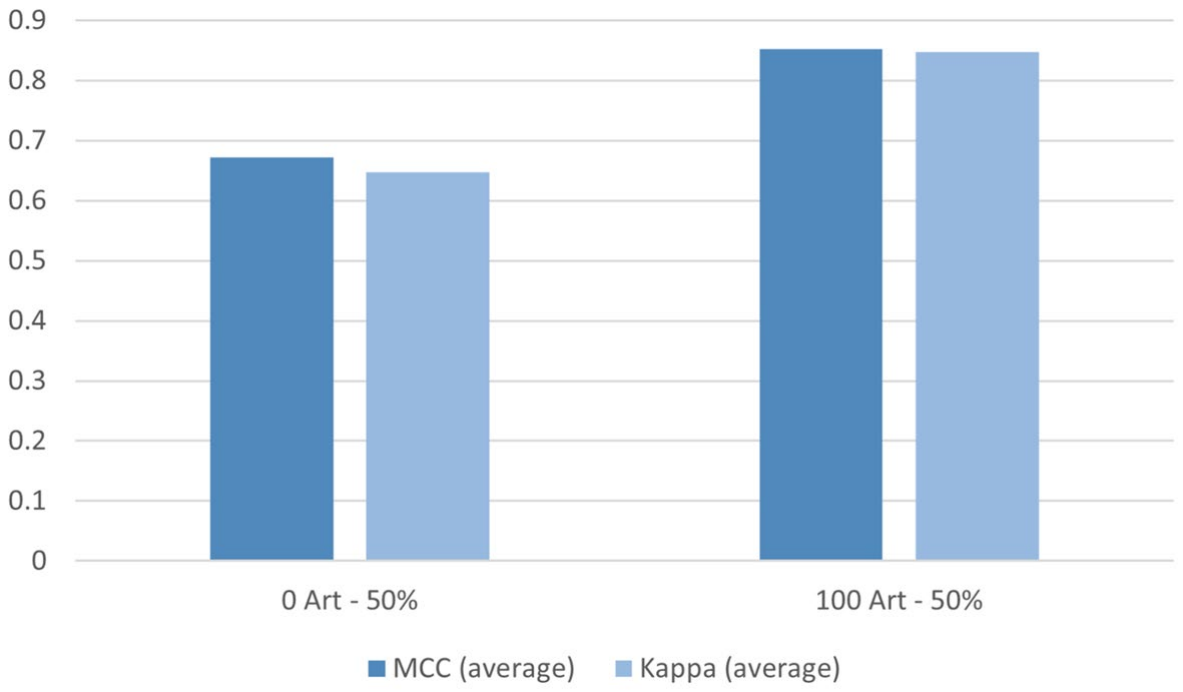
Mean values for MCC and Kappa, for the case of no artificial frames (left) and 100 artificial frames per group (right). In both cases, 50% of the real data is used for training.

The results for the best case (100 artificial samples per group and 50% of real samples for training) are highlighted in Fig. 10. We can see that the two indices are positive values and close to 1. According to^19^, this indicates a high capacity to detect positive and negative cases with a minimum error. Furthermore, as detailed in^19^, the case where both indices show a very similar positive result correspond to a well-balanced experiment. Comparing the best case with the same configuration without the use of artificial samples, an improvement of about 20% is observed in both the MCC and the Kappa index.

It is also important to mention that continuously increasing the percentage of artificial samples do not improve the results, as a saturation effect appears. This is evidenced by the results obtained when using 250 artificial samples in all the experiments, which are similar to the ones obtained with only 50 artificial samples (see Fig. 7).

If we compare the best result obtained with the strategy proposed in this paper with those already published in other papers, we can see that the method proposed here is powerful since only using the X and Y coordinates achieves 92.40% accuracy, which is better than the 91% obtained in^5^ using 84 linear and non-linear features extracted from all the available variables generated by the digitizer tablet; and similar to the value obtained in^6^ using 77 linear and non-linear features. However, our method is below the 97.96% reported in^7^, which obtained this result after selecting the best 5 features over a set of 35 pre-computed linear and non-linear features (in time and frequency domains) and combining two different strategies (residual method and radius method). However, this is a complex system based on the pre-calculation of a large set of features, in contrast to the proposed system using LSTM, where no features are calculated beforehand.

Finally, in terms of the computational time needed by the system, all DA methods required much less time compared to the time used to train the LSTM model. In a desktop Intel(R) Core(TM) i7-7700K CPU @ 4.20 GHz with 16.0 GB of RAM, the training of the LSTM network took about 75 min, while the DA methods took only a few seconds to generate the artificial samples. The only two methods that took slightly longer were the mEMD method, which took about 5 min for the decomposition of the time series, and the SPAWNER method, which took about 10 min. Therefore, if we are interested in reducing the overall computational time, efforts should be made to reduce the time needed for training the model.

## Conclusion

This study presents a deep learning LSTM network for the diagnosis of essential tremor from the X and Y coordinates of the Archimedes spiral. Since deep neural networks need a large amount of data to converge, a data augmentation strategy based on a decomposition–recombination strategy using mEMD was implemented. The artificial samples avoided overfitting in the training stage of the LSTM network and increased the classification results in the test stage. Moreover, since the acquisition of the original data required specific equipment, the study was carried out using only the X and Y coordinate points, with the idea that it can be easily replicated by acquiring the data using any digitising tablet.

The results obtained with the proposed method show a significant improvement of about 10% compared to the case without artificial samples. The other DA methods only showed an improvement of half or less than that achieved by our decomposition–recombination method, and even worsened the result in 4 cases. Furthermore, the proposed method has proven to be useful when only 30% of the available samples were used for training, which is an interesting result when dealing with small datasets.

These results have been produced by generating 600–800% more artificial samples than the existing number of original samples. In the case of increasing the number of artificial samples above these percentages, the results obtained are still better than when using only real samples, but at the same time do not improve on or worsen the results achieved when fewer artificial samples are used. This effect could be explained by the fact that excessive reuse of the original data to create more artificial samples causes a saturation effect when training the model.

In future works, faster methods for decomposing the samples with mEMD, such as serial-EMD^20^, could be explored. Taking into account that the decomposition–recombination strategy works well, other types of decomposition methods could also be investigated. For example, the DCT transform was used in^18^ to decompose iEEG signals in frequency bands and new artificial data was created by recombining frequency bands from different subjects, which could also be used with handwritten signals. Another aspect that could be further investigated would be to add attention layers in the LSTM network, which could improve the performance of the model. In that sense, Transformers^21^ could be a good choice for use in this application. Finally, we would like to stress that the use of this approach could benefit the advancement of low-cost, non-invasive methods that are easily adaptable to patients and hospitals, as they can be useful and helpful in real-world situations from a social and economic point of view.

## Data Availability

The datasets generated by and/or analyzed during the current study are not publicly available due to ethics and privacy requirements, but they are available from the corresponding author upon reasonable request.

## Code availability

The code for creating artificial samples using EMD is available at https://github.com/ffbear1993/DR-EMD. The serialisation method to speed the EMD decoposition (serial-EMD) is available at https://github.com/ffbear1993/serial-emd. The code for the other data augmentation methods is available at https://github.com/uchidalab/time_series_augmentation.
